# High Estimated 24‐Hour Urinary Sodium Excretion is Associated With Metabolic Syndrome and Its Phenotypes: Tehran Lipid and Glucose Study

**DOI:** 10.1155/jnme/1819496

**Published:** 2026-07-14

**Authors:** Niloufar Rasaei, Zahra Bahadoran, Fereidoun Azizi

**Affiliations:** ^1^ Micronutrient Research Center, Research Institute for Endocrine Disorders, Research Institute for Endocrine Sciences, Shahid Beheshti University of Medical Sciences, Tehran, Iran, sbmu.ac.ir; ^2^ Endocrine Research Center, Research Institute for Endocrine Disorders, Research Institute for Endocrine Sciences, Shahid Beheshti University of Medical Sciences, Tehran, Iran, sbmu.ac.ir

**Keywords:** cardiometabolic risk factors, metabolic syndrome, salt, urinary sodium excretion

## Abstract

**Aim:**

This study aimed to investigate the association between estimated 24‐h urinary sodium (24 h‐UNa) excretion and metabolic syndrome (MetS) prevalence and its components in adults.

**Methods:**

This cross‐sectional study (2015–2017) included 2057 men and women (mean age: 45.5 ± 14.8 years; 45.5% men). Anthropometric assessments and biochemical measurements were conducted according to standard protocols. Na intake was estimated from second‐void morning urine specimens using the Tanaka formula, which incorporates spot UNa, potassium (UK), and creatinine (UCr) levels. MetS was defined based on the National Cholesterol Education Program Adult Treatment Panel III (NCEP‐ATP III) criteria, encompassing hyperglycemia (G), hypertriglyceridemia (T), low high‐density lipoprotein cholesterol (HDL‐C) (H), hypertension (B), and abdominal obesity (W). Multivariable logistic regression, adjusted for age, sex, estimated glomerular filtration rate (eGFR), physical activity level (PAL), and smoking status, was employed to calculate odds ratios (ORs) and 95% confidence intervals (CIs) for MetS and its components across tertiles of 24 h‐UNa (< 124, 124–162, and ≥ 162 mmol/day).

**Results:**

The overall MetS prevalence was 37.6%, with a mean estimated 24 h‐UNa excretion of 3491 ± 1020 mg/day. After multivariable adjustment, high UNa excretion was independently associated with increased odds of MetS (OR = 1.49, 95% CI = 1.16–1.92), abdominal obesity (OR = 2.70, 95% CI = 2.09–3.49), and hypertriglyceridemia (OR = 1.48, 95% CI = 1.16–1.89). However, no significant association was observed between high UNa excretion and hypertension (OR = 1.13, 95% CI = 0.86–1.48). Furthermore, the prevalence of specific MetS phenotypes was significantly elevated in the highest compared to the lowest 24 h‐UNa tertile (e.g., WBH [13.3% vs. 11.1%, *p* = 0.018], WBT [18.5% vs. 14.1%, *p* = 0.033], BHT [11.3% vs. 10.5%, *p* = 0.028], WHT [22.1% vs. 17.7%, *p* = 0.006], and WBHT [10.4% vs. 9.3%, *p* = 0.019]).

**Conclusion:**

Elevated estimated 24 h‐UNa excretion is independently associated with a higher likelihood of MetS and its key components, particularly abdominal obesity and hypertriglyceridemia. These findings suggest that high dietary Na intake may be an independent risk factor for MetS, and that dietary salt reduction could play a vital role in the primary prevention of MetS.

## 1. Introduction

Metabolic syndrome (MetS) represents various cardiometabolic risk factors that collectively increase susceptibility to different chronic diseases and increase all‐cause mortality risk [1–3]. The global prevalence of MetS varies (17%–43%), with a pronounced age‐dependent progression exceeding 42% in adults aged more than 60 years [4, 5]. The pathophysiology of MetS risk shows complex interactions between nonmodifiable and modifiable factors, particularly unhealthy dietary patterns with high sodium (Na) intake [6–8].

While the hypertensive effects of Na are established, growing evidence suggests its broader metabolic consequences [9, 10]. Some previous studies showed the association between high levels of urinary Na (UNa) or UNa to potassium (UK) ratio and hypertriglyceridemia and MetS risk [5, 10, 11]. Furthermore, evidence from normotensive populations indicates that high UNa excretion is associated with characteristics of MetS, including elevated blood pressure (BP) and obesity [12]. As well as, recent evidence has shown that higher UNa to UK ratio excretion is independently associated with increased heart disease and myocardial infarction (MI) risk [13]. Moreover, emerging data suggest that high UNa excretion may contribute to the pathogenesis of nonalcoholic fatty liver disease (NAFLD), further expanding the metabolic consequences of excessive salt consumption [14]. However, the body of evidence remains inconsistent, reporting null [15] or potentially detrimental associations [10, 16, 17], which underscores significant knowledge gaps. Findings are inconsistent due to methodological heterogeneity in study designs, durations of intervention, and baseline characteristics of participants [11, 15–17].

While the 24‐h urine collection is the gold standard, its practicality and cost are prohibitive for large‐scale epidemiological studies [18, 19]. The use of validated estimation equations, such as the Tanaka formula [20], which has been calibrated in various populations, including our Iranian cohort [21], provides a feasible and acceptable alternative for population‐level screening. Therefore, this study aimed to investigate the association between estimated 24‐h urinary sodium (24 h‐UNa) excretion (using the Tanaka formula) and MetS and its distinct phenotypes in Iranian adults participating in a nationally representative population. This approach allows us to explore this association using a more objective biomarker of Na intake than dietary recalls. We hypothesize that 24 h‐UNa excretion may have associations with MetS risk and phenotypes.

## 2. Methods

### 2.1. Study Population

This study was conducted within the framework of the Tehran Lipid and Glucose Study (TLGS), a large‐scale, population‐based cohort encompassing 15,005 individuals residing in District 13 of Tehran, Iran. Established in 1999, the TLGS performs follow‐up assessments at three‐year intervals [22]. For the present analysis, a subset of 2057 adult participants (aged 19–92 years; 45.5% male) from the sixth TLGS examination phase (2015–2017) was included. Eligibility criteria required complete datasets on urinary biomarkers (Na, K, creatinine [Cr]) and anthropometric, biochemical, and clinical measurements. The research protocol complied with the ethical guidelines outlined in the Declaration of Helsinki. It was approved by the Ethics Committee of the Research Institute for Endocrine Sciences at Shahid Beheshti University of Medical Sciences (Ethics Code: IR.SBMU.ENDOCRINE.REC.1404.021). Prior to enrollment, written informed consent was secured from all participants.

### 2.2. Data Collection and Measurements

The TLGS adheres to standardized data collection protocols, with comprehensive methodological details published elsewhere [22–24]. A comprehensive description of demographic characteristics, anthropometric measurements, and biochemical parameters has been previously published [25]. Certified interviewers gathered updated data on smoking habits, medication use, and physical activity level (PAL) through the use of validated questionnaires. Anthropometric assessments were conducted in accordance with the World Health Organization (WHO) guidelines, and body mass index (BMI) was derived from these measurements. BP readings, including systolic BP (SBP) and diastolic BP (DBP), were obtained after a 10‐min rest period using calibrated sphygmomanometers. For biochemical evaluations, participants fasted for 12–14 h prior to blood sampling, and fasting serum glucose (FSG), triglyceride (TG), and high‐density lipoprotein cholesterol (HDL‐C) were measured. Individuals not receiving hypoglycemic treatment underwent a 75‐g oral glucose tolerance test (82.5 g glucose monohydrate; Cerestar EP, Spain), with 2 h postload glucose measurements [23]. All laboratory analyses were conducted at the centralized TLGS facility using Pars Azmoon assay kits (Tehran, Iran) on Selectra 2 automated analyzers (Vital Scientific, Netherlands), following stringent quality control procedures. The analytical precision of all assays was confirmed, with both interassay and intraassay coefficients of variation (CVs) remaining below 5%.

### 2.3. Estimated Glomerular Filtration Rate (eGFR) Calculation

The eGFR was calculated using the CKD‐EPI equation [24], standardized to body surface area and reported in mL/min/1.73 m^2^.

### 2.4. Estimation of 24 h‐UNa

Fasting second‐void morning urine specimens were collected after a 10–12 h overnight fast. Spot UNa and K (UK) concentrations were quantified using flame photometry (Screen Lyte system, Hospitex Diagnostics, Italy), demonstrating high precision with both intra‐ and interassay CVs below 5%. To evaluate biological variability, a reliability subset (*n* = 13) provided repeated samples at a two‐week interval. Urinary Cr (UCr) and serum Cr (SCr) were assayed via the Jaffe reaction, which exhibited comparable reproducibility, with quality control results confirming intra‐ and interassay CVs consistently under 5%. The Tanaka equation [20], previously validated in our study population [21], was applied to estimate 24 h‐UNa excretion (mmol/day). The sex‐inclusive formula is expressed as follows: Estimated 24 h‐UNa = 23 × 21.98 × (0.1 × [−2.04 × age + 14.89 × weight + 16.14 × height − 2244.45] × Spot UNa ÷ Spot UCr)^0.392^.

### 2.5. MetS and Its Components

MetS was diagnosed based on the harmonized definition proposed by Alberti et al. (Circulation, 2009) [26], which requires the presence of ≥ 3 of the following 5 metabolic abnormalities. Consistent with this harmonized statement, which permits population‐ and country‐specific waist circumference thresholds [27], we used modified waist circumference cutoffs specific to Iranian adults as recommended by the Iranian National Committee of Obesity: (1) Hyperglycemia: FSG ≥ 100 mg/dL (5.6 mmol/L) or current use of glucose‐lowering medication; (2) hypertriglyceridemia: serum TG ≥ 150 mg/dL (1.69 mmol/L) or lipid‐lowering treatment; (3) low HDL‐C: serum HDL‐C < 40 mg/dL (1.04 mmol/L) in men or < 50 mg/dL (1.29 mmol/L) in women, or relevant pharmacotherapy; (4) hypertension: BP ≥ 130/85 mmHg or antihypertensive medication use; and (5) abdominal obesity: waist circumference ≥ 95 cm (applied uniformly to both sexes). MetS phenotypes were systematically categorized using an alphanumeric coding system based on combinations of the five diagnostic components. Each phenotype was designated by a unique letter code representing the specific combination of metabolic abnormalities present: W: abdominal obesity; G: hyperglycemia; T: hypertriglyceridemia; B: elevated BP; H: low HDL‐C. All possible combinations of three or more components were considered distinct phenotypic variants of MetS.

### 2.6. Statistical Methods

Data were analyzed using SPSS (Version 26.0; IBM Corporation, USA) and GraphPad Prism (Version 8; USA), with statistical significance defined as a two‐tailed *p* value < 0.05. Continuous variables are expressed as mean ± standard deviation (SD), while categorical variables are reported as percentages. Baseline characteristics across tertiles of estimated 24 h‐UNa excretion were compared using one‐way ANOVA for continuous variables or chi‐square tests for categorical variables. The relationships between 24 h‐UNa tertiles and MetS and its components were evaluated using binary logistic regression, and three models were conducted: Model 1 (adjusted for age and sex), Model 2 (additionally adjusted for eGFR), and Model 3 (further adjusted for PAL and smoking). The stepwise adjustment approach was considered to sequentially control for confounders based on their biological and behavioral nature. Model 1 adjusted for demographic factors (age and sex). Model 2 additionally adjusted for eGFR, a physiological confounder reflecting renal function that directly impacts UNa excretion and is independently associated with BP and MetS. Model 3 further adjusted for PAL and smoking status as behavioral or lifestyle confounders. This hierarchical strategy allowed us to assess the effect of renal function (Model 2) before accounting for modifiable lifestyle behaviors (Model 3), thereby clarifying the independent contribution of UNa excretion to MetS risk. The distribution of MetS phenotypes across tertiles of estimated 24 h‐UNa excretion was evaluated using GraphPad Prism software.

## 3. Results and Discussion

### 3.1. Results

The study included 2057 participants (mean age: 45.5 ± 14.8 years; 45.5% male). The mean estimated 24 h‐UNa excretion, derived using Tanaka’s equation, was 3491 ± 1020 mg/day. The prevalence of MetS was estimated at 37.6%. As shown in Table 1, participants in the highest tertile of UNa excretion exhibited significantly higher values compared to the lowest tertile for SBP (115 vs. 105 mm Hg), DBP (76.5 vs. 71.5 mm Hg), BMI (28.9 vs. 25.8 kg/m^2^), WC (95.1 vs. 86.2 cm), TG (146 vs. 130), SCr concentration (1.07 vs. 1.03 μmol/L), spot UNa (181 vs. 90.8 mmol/L), and estimated 24 h‐UNa excretion (194 vs. 100 mmol/day) (*p* for all < 0.050). Although spot UCr (119 vs. 238 mmol/L) and HDL‐C (46.1 vs. 48.6 mmol/L) were lower in the highest vs. the lowest tertile (*p* for both < 0.001).

**TABLE 1:** Characteristics of the study participants in tertiles of estimated 24‐h urinary sodium excretion.

	Estimated 24‐h urinary sodium excretion			
	< 124	124–162	≥ 162	*p* value
Age (year)	37.3 ± 14.9	39.7 ± 15.2	41.916.6	0.011
Men (%)	37.5	46.4	61.2	0.001
Glucose‐lowering drugs (%)	6.3	7.7	8.1	0.491
Lipid‐lowering drugs (%)	10.4	11.5	6.7	0.173
Blood pressure–lowering drugs (%)	9.7	11.1	8.6	0.729
Current smoker (%)	14.1	11.9	17.9	0.472
Occupational status				0.002
Employed (%)	40.1	50.7	50.7	
Unemployed (%)	1.3	1.5	0.9	
Housewife (%)	41.1	29.9	27.9	
Educational certificate				0.001
Illiterate + primary (%)	11.8	9.0	12.2	
Secondary + diploma (%)	43.7	51.2	53.3	
Higher diploma (%)	44.5	39.9	34.5	
SBP (mm Hg)	105 ± 16.3	111 ± 18.7	115 ± 18.3	0.001
DBP (mm Hg)	71.1 ± 12.8	75.3 ± 11.7	76.5 ± 10.9	0.001
BMI (kg/m^2^)	25.8 ± 4.99	27.0 ± 5.10	28.9 ± 6.97	0.001
WC (cm)	86.2 ± 12.2	90.8 ± 12.8	95.1 ± 13.5	0.001
FSG (mg/dL)	97.1 ± 27.9	100 ± 33.7	99.7 ± 22.7	0.516
2h‐SG (mg/dL)	103 ± 36.0	110 ± 54.6	105 ± 32.9	0.445
HDL‐C (mg/dL)	48.6 ± 10.9	46.0 ± 11.3	46.1 ± 10.7	0.001
TG (mg/dL)	130 ± 89.3	143 ± 83.2	146 ± 83.6	0.001
PAL (%)				0.364
Low	11.8	9.0	12.2	
Moderate	43.7	51.2	53.3	
High	44.5	39.9	34.5	
Serum creatinine (μmol/L)	1.03 ± 0.15	1.04 ± 0.16	1.07 ± 0.17	0.022
eGFR (mL/min per 1.73 m^2^)	79.8 ± 18.7	80.2 ± 18.3	79.2 ± 17.3	0.859
Spot urinary sodium (mmol/L)	90.8 ± 44.4	139 ± 51.4	181 ± 50.3	0.001
Spot urinary potassium (mmol/L)	73.2 ± 36.4	74.3 ± 39.5	68.1 ± 30.3	0.163
Spot urinary creatinine (mmol/L)	238 ± 102	171 ± 73.3	119 ± 50.5	0.001
Estimated 24‐h UNa^∗^ (mmol/day)	100 ± 18.1	142 ± 11.3	194 ± 28.0	0.001

*Note:* Data are mean ± SD or percent. One‐way ANOVA and chi‐square test were used for continuous and categorical variables, respectively. TG, serum triglyceride.

Abbreviations: 2h‐SG, 2h serum glucose; BMI, body mass index; DBP, diastolic blood pressure; eGFR, estimated glomerular filtration rate; FSG, fasting serum glucose; HDL‐C, high‐density lipoprotein cholesterol; PAL, physical activity level; SBP, systolic blood pressure; WC, waist circumference.

^∗^Estimated 24 h sodium excretion calculated from Tanaka’s equation.

Table 2 presents odds ratios (ORs) and confidence intervals (CIs) for the association between estimated 24 h‐UNa excretion tertiles and MetS surrogates. Participants in the highest UNa excretion tertile (≥ 162 mmol/day) had a significantly higher probability of MetS after adjusting for age, sex, eGFR, PAL, and smoking (OR = 1.49, 95% CI = 1.16–1.92). Similarly, the highest UNa tertile was associated with a markedly increased probability of abdominal obesity in fully adjusted Model 3 (OR = 2.70, 95% CI = 2.09–3.49). In addition, participants in the highest tertile demonstrated a significantly higher likelihood of elevated serum TG levels in fully adjusted Model 3 (OR = 1.48, 95% CI = 1.16–1.89). In contrast, no significant associations were observed between the highest estimated 24 h‐UNa excretion tertile and serum HDL‐C, FSG, or BP in either crude or adjusted models after accounting for potential confounders.

**TABLE 2:** The odds ratios (ORs) and 95% confidence intervals (CIs) of metabolic syndrome and its components across tertiles of estimated 24 h urinary sodium excretion.

	Estimated 24‐h urinary sodium excretion^∗^		
	< 124	124–162	≥ 162
Metabolic syndrome			
Crude	1.00	1.45 (1.16–1.81)	1.52 (1.22–1.91)
Model 1	1.00	1.48 (1.16–1.89)	1.52 (1.19–1.94)
Model 2	1.00	1.46 (1.14–1.87)	1.49 (1.16–1.90)
Model 3	1.00	1.42 (1.10–1.82)	1.49 (1.16–1.92)
Abdominal obesity			
Crude	1.00	1.80 (1.45–2.24)	2.74 (2.18–3.44)
Model 1	1.00	1.77 (1.39–2.25)	2.71 (2.11–3.48)
Model 2	1.00	1.77 (1.39–2.52)	2.68 (2.08–3.44)
Model 3	1.00	1.76 (1.38–2.24)	2.70 (2.09–3.49)
Low HDL‐C			
Crude	1.00	1.01 (0.81–1.25)	0.82 (0.66–1.02)
Model 1	1.00	1.10 (0.88–1.37)	0.91 (0.79–1.13)
Model 2	1.00	1.09 (0.87–1.36)	0.89 (0.72–1.12)
Model 3	1.00	1.08 (0.86–1.35)	0.91 (0.73–1.14)
High serum triglycerides			
Crude	1.00	1.54 (1.23–1.91)	1.61 (1.29–2.01)
Model 1	1.00	1.46 (1.15–1.85)	1.48 (1.17–1.88)
Model 2	1.00	1.45 (1.14–1.84)	1.45 (1.14–1.84)
Model 3	1.00	1.48 (1.16–1.89)	1.48 (1.16–1.89)
High fasting blood glucose			
Crude	1.00	1.11 (0.87–1.41)	1.03 (0.80–1.31)
Model 1	1.00	1.10 (0.85–1.43)	0.99 (0.76–1.29)
Model 2	1.00	1.09 (0.83–1.42)	0.97 (0.74–1.27)
Model 3	1.00	1.07 (0.82–1.40)	0.93 (0.71–1.22)
Hypertension			
Crude	1.00	1.27 (1.01–1.60)	1.19 (0.94–1.50)
Model 1	1.00	1.26 (0.97–1.24)	1.12 (0.86–1.45)
Model 2	1.00	1.25 (0.96–1.62)	1.10 (0.84–1.43)
Model 3	1.00	1.25 (0.96–1.62)	1.13 (0.86–1.48)

*Note:* Data are odds ratios and 95% CIs. Binary logistic regression test was used (tertile of 1 was considered as reference). Model 1, adjusted for age and sex; Model 2, adjusted for age, sex, and eGFR; Model 3, adjusted for age, sex, eGFR, PAL, and smoking.

Abbreviations: eGFR, estimated glomerular filtration rate; HDL‐C, high‐density lipoprotein cholesterol; PAL, physical activity level.

^∗^Estimated 24 h sodium excretion calculated from Tanaka’s equation.

**FIGURE 1:**
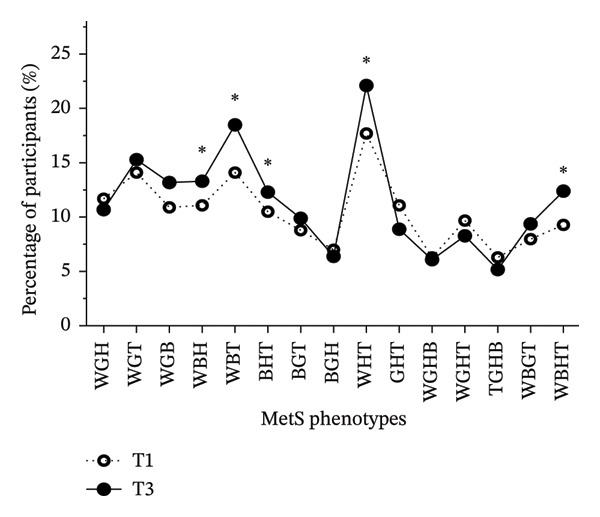
The prevalence of MetS phenotypes across tertiles of estimated 24 h‐UNa excretion. MetS, metabolic syndrome; W, elevated waist circumference; G, elevated blood glucose; T, elevated triglyceride levels; B, elevated blood pressure; H, low‐high‐density lipoprotein cholesterol; T1, first tertile (< 124 mmol/day); T3, third tertile (≥ 162 mmol/day); 24 h‐UNa, 24 h urinary sodium. ^∗^ *p* < 0.05 for the following phenotypes: WBH, WBT, BHT, WHT, and WBHT (comparison between the lowest (T1) and highest (T3) tertiles of estimated 24 h‐UNa excretion). All other comparisons were not statistically significant. Data are presented as prevalence (%).

The prevalence of MetS phenotypes across tertiles of estimated 24 h‐UNa excretion is illustrated in Figure 1. Subjects in the highest (≥ 162 mmol/day) compared to the lowest tertile (< 124 mmol/day) had a significantly higher prevalence of WBH (13.3% vs. 11.1%, *p* = 0.018), WBT (18.5% vs. 14.1%, *p* = 0.033), BHT (11.3% vs. 10.5%, *p* = 0.028), WHT (22.1% vs. 17.7%, *p* = 0.006), and WBHT (10.4% vs. 9.3%, *p* = 0.019) phenotypes.

## 4. Discussion

The result of this study showed a significant positive association between elevated 24 h‐UNa excretion (≥ 162 mmol/day) and abdominal obesity, hypertriglyceridemia, and MetS prevalence in Iranian adults. This association suggests that excessive Na intake may contribute to the pathogenesis of metabolic disorders. To our knowledge, this represents the first population‐based study examining the association between estimated 24 h‐UNa excretion and MetS and its components in an Iranian adult population.

Current studies show that high Na consumption is associated with an increased risk of metabolic disorders, including insulin resistance (IR), adiposity, hypertension, CVD, and Type 2 diabetes (T2D) [28–31]. However, the mechanistic association between Na status and MetS risk remains unclear, with studies reporting conflicting findings such as inverse [16, 17], null [15, 32], or potentially detrimental associations [2, 5, 6, 11]. A meta‐analysis involving 51,886 participants demonstrated that individuals with high UNa excretion had a 37% increased risk of developing MetS [11]. However, this analysis relied on self‐reported dietary assessments (e.g., food records, 24 h recalls, and food frequency questionnaires), which increase the underestimation of true Na intake [11]. While 24 h urine collections remain the gold standard for Na assessment, they have high cost and participant burden, which limit sample sizes in studies [33, 34]. Notably, a Korean study using spot urine samples reported significant associations between high Na intake and MetS components [35], whereas others found links only to central obesity and hypertension, with no significant relationship to dyslipidemia or blood glucose [36, 37]. These discrepancies align with prior studies employing 12 h or 24 h urine collections [1, 33]. Our findings demonstrate positive associations between 24 h‐UNa excretion and central obesity, hypertriglyceridemia, and MetS prevalence; interestingly, one population‐based study reported a dose‐dependent association between higher Na excretion and MetS prevalence, particularly central obesity and elevated BP [33].

The precise mechanisms linking Na intake to MetS remain incompletely understood, underscoring the need for well‐designed longitudinal studies with standardized, repeated 24 h UNa measurements [28]. Central obesity and IR, as core features of MetS, may promote Na retention and extracellular fluid expansion, potentially exacerbating the BP response to dietary Na [11]. Obesity also plays an important role in hypertension, with IR mediating this pathogenic link to elevated BP [38]. Epidemiological evidence shows that increased UNa excretion and dietary Na intake may independently predict visceral adiposity, body fat percentage, and obesity incidence [39, 40]. Excessive Na intake may increase adipogenesis by upregulating lipogenic activity and subsequent fat deposition [11]. Moreover, metabolic disturbances induced by Na intake may impair glucose homeostasis, compounded by visceral adiposity and elevated free fatty acid release [41]. The association between MetS and Na homeostasis involves modulation of renal Na transport [42]. Experimental evidence demonstrates that insulin increases renal Na reabsorption via Na–hydrogen exchanger type 3 (NHE3) upregulation [43, 44], enhances basolateral Na efflux through Na bicarbonate cotransporter (NBCe1) activation [44], and stimulates Na/K‐ATPase activity across nephron segments through the Phosphoinositide 3‐kinase (PI3K)/3‐Phosphoinositide‐dependent protein kinase 1 (PDK1) pathway [11, 45]. Beyond these classic pathways, emerging evidence has identified several novel mechanisms. First, high Na intake activates salt‐inducible kinases (SIKs) in adipocytes, which regulate insulin signaling, glucose uptake, lipogenesis, and thermogenesis; dysregulation of SIKs contributes to adipose tissue dysfunction and metabolic inflammation [46]. Second, excessive Na stimulates the aldose reductase–fructokinase pathway in both the liver and hypothalamus, leading to increased endogenous fructose production. This metabolic shift induces leptin resistance, impairs satiety signaling, disrupts appetite regulation, and promotes hepatic fat accumulation independent of caloric intake [47]. Third, in the context of obesity, high Na intake exacerbates oxidative stress and disrupts renal Na metabolism via Na/K‐ATPase signaling; the antioxidant enzyme Heme oxygenase‐1 (HO‐1) modulates this pathway and may represent a therapeutic target [48]. Notably, high Na diets often coincide with excessive caloric intake from processed foods [49]. However, emerging evidence suggests that Na may directly contribute to adipocyte hypertrophy and metabolic dysfunction independent of caloric intake [50, 51]. These findings underscore the necessity for further research to elucidate the distinct role of Na in MetS pathogenesis.

This study has several strengths, including its large, population‐based design, the use of an objective biomarker (estimated 24 h‐UNa) superior to self‐reported dietary recalls, and its status as the first investigation of this relationship in a representative Iranian adult population, providing valuable insights for this population. However, several limitations must be acknowledged. First, the cross‐sectional nature of our analysis prevents the establishment of causal relationships. Second, although we adjusted for several potential confounders, we did not have comprehensive data on overall dietary quality, such as total caloric intake, dietary patterns, or consumption of processed foods. High Na intake is often correlated with poor dietary quality, and we cannot rule out the possibility of residual confounding by these unmeasured dietary factors. Therefore, the observed associations may partially reflect the effect of an overall unhealthy dietary pattern rather than Na alone. Third, the homogeneous ethnic composition of our study may limit the generalizability of our findings to other ethnic groups. Finally, our reliance on a single spot urine sample to estimate 24 h excretion, while a feasible necessity given the low compliance and high burden of 24 h collections in large epidemiological studies [51–54], introduces potential measurement error. Nevertheless, we employed second‐morning void samples, which demonstrate a better correlation with 24 h excretion than random spot samples [55, 56], and utilized the Tanaka formula, which has been previously validated in our population [21], thereby enhancing the reliability of our estimations for group‐level analyses.

## 5. Conclusion

This study demonstrates a significant association between higher 24 h‐UNa excretion and increased prevalence of MetS, particularly its components of abdominal obesity and hypertriglyceridemia, in a large sample of Iranian adults. These results underscore the potential role of excessive dietary salt intake as a modifiable risk factor for MetS. Consequently, our study provides evidence to support the incorporation of dietary Na reduction into public health strategies for the prevention and management of MetS. Future research should employ longitudinal designs and randomized controlled trials to establish this relationship.

## Funding

This research received no specific grant from any funding agency in the public, commercial, or not‐for‐profit sectors.

## Conflicts of Interest

The authors declare no conflicts of interest.

## Data Availability

Access to study data is available upon reasonable request by contacting the corresponding author, subject to approval by the RIES director (azizi@sbmu.ac.ir).
